# Megabladder mouse model of congenital obstructive nephropathy: genetic etiology and renal adaptation

**DOI:** 10.1007/s00467-013-2658-6

**Published:** 2013-11-26

**Authors:** Kirk M. McHugh

**Affiliations:** 1 Department of Pediatrics and Division of Anatomy, College of Medicine, The Ohio State University, Columbus, OH 43210 USA; 2 Center for Molecular and Human Genetics, The Research Institute, Nationwide Children’s Hospital, Columbus, OH 43205 USA

**Keywords:** Congenital obstructive nephropathy, Kidney pathogenesis, Animal models of disease, Long-range enhancer

## Abstract

Congenital obstructive nephropathy remains one of the leading causes of chronic renal failure in children. The direct link between obstructed urine flow and abnormal renal development and subsequent dysfunction represents a central paradigm of urogenital pathogenesis that has far-reaching clinical implications. Even so, a number of diagnostic, prognostic, and therapeutic quandaries still exist in the management of congenital obstructive nephropathy. Studies in our laboratory have characterized a unique mutant mouse line that develops in utero megabladder, variable hydronephrosis, and progressive renal failure. Megabladder mice represent a valuable functional model for the study of congenital obstructive nephropathy. Recent studies have begun to shed light on the genetic etiology of *mgb* ^*−/−*^ mice as well as the molecular pathways controlling disease progression in these animals.

## Introduction

One key component in studying the complex processes involved in the pathogenesis of human disease is the use of animal models. Whether experimental, surgical or genetic, animal models provide a wealth of information on how pathogenic processes affect the whole organism in the context of dynamic pathophysiological responses. Gaining a better understanding of the etiology and progression of a disease is critical in determining the precise prognostic and therapeutic strategies needed to stage, treat, and cure the disease. In this review, we will examine two key elements of urogenital pathogenesis associated with the mgb mouse model of congenital obstructive nephropathy (CON): genetic etiology and renal pathogenesis.

## Genetic etiology

### The mgb mouse model

Homozygotic *mgb* mice (*mgb* ^*−/−*^) develop lower urinary tract obstruction in utero due to a primary defect in bladder smooth muscle differentiation [1]. This defect is the result of a random transgene insertion into chromosome 16 and translocation of a fragment of chromosome 16 containing the transgene into chromosome 11. Genetic studies indicate that the transgene plays no biological role in generating the mgb phenotype [1]. In addition, although transcriptional profiling of *mgb* ^*−/−*^ mice identified an over-expressed cluster of three genes on the translocated fragment of chromosome 16, none of these transcripts plays a direct role in generating the mgb phenotype [2].

These observations suggest that the primary genetic defect associated with the *mgb* ^*−/−*^ phenotype resides on chromosome 11. Unpublished results indicate that the translocation breakpoint on chromosome 11 occurs approximately 500 kb upstream of a key transcription factor associated with smooth muscle development—myocardin. Complementation and expression studies have confirmed that the gene responsible for the *mgb* ^*−/−*^ phenotype is myocardin and that this gene plays no role in normal kidney development or function [2].

*Mgb* ^*−/−*^ mice develop a nonfunctional, over-distended bladder that most closely resembles a non-neurogenic neurogenic bladder. Affected animals develop low-pressure hydronephrosis that initiates in utero, producing a functional lower urinary tract obstruction, antenatal hydronephrosis, and signs of renal failure evident shortly after birth [1]. *Mgb* ^−/−^ mice are born with histopathological evidence of renal injury and exhibit a variable clinical course similar to children with posterior urethral valves (PUV). In addition, affected animals preferentially develop unilateral, right-sided hydronephrosis reminiscent of the “pop-off” mechanism theorized in children with PUV and secondary unilateral vesicoureteral reflux [3]. Finally, *mgb* ^−/−^ mice can be rescued from the complications of renal failure by cutaneous vesicostomy even though 40 % die within the first 2 weeks despite a patent stoma and no apparent surgical complications, a result reminiscent of the fact that 27 % to 70 % of children with PUV will have progressive chronic kidney disease despite surgery [4–6].

### Patent ductus arteriosus

**Fig. 1:**
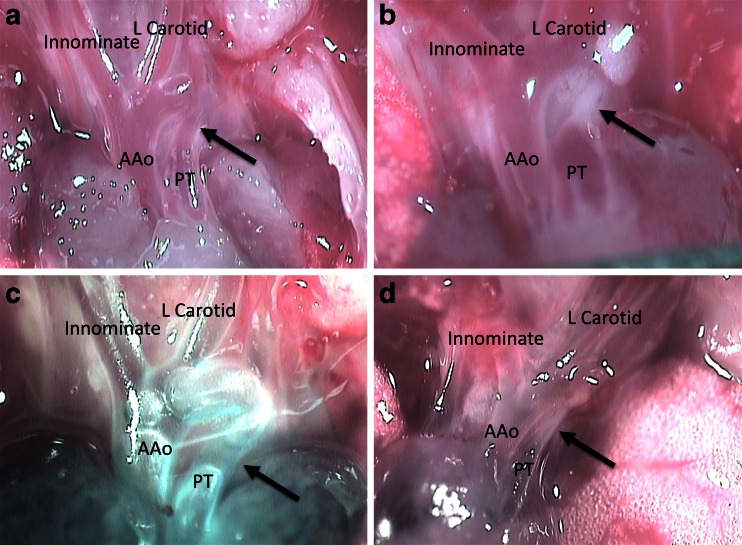
Postnatal day 2 outflow tracts** a**,** b** without and** c**,** d** with methylene blue injection showing patent ductus arteriosus in the *mgb* compound heterozygote (**a**, **c**; *arrow*) versus ligamentum arteriosum in the control (**b**, **d**; *arrow*). Left carotid artery (*L Carotid*), innominate (brachiocephalic) artery (*Innominate*), ascending aorta (*AAo*), and pulmonary trunk (*PT*)

Recent studies indicate that further reduction in myocardin expression through genetic manipulation not only recapitulates the *mgb* ^*−/−*^ bladder phenotype, but also results in the appearance of a second genetic defect—patent ductus arteriosus (Fig. 1). Although a direct link between bladder smooth muscle development and patent ductus arteriosus may not be self-evident, a review of their developmental origins identifies a common cellular lineage. During cardiac development, the outflow tract receives a critical contribution from the cranial neural crest associated with the branchial arches. These cells seed the developing cardiac outflow tract and its associated vessels providing them with the smooth muscle progenitors necessary for normal vascular development. Even though bladder smooth muscle is principally derived from splanchnic mesenchyme (mesoderm) and not neural crest (neuroectoderm), the smooth muscle differentiation program in both cell types is controlled by myocardin expression. Morphological analysis confirmed a lack of smooth muscle cells within the ductus arteriosus of these animals (unpublished results). Therefore, the appearance of patent ductus arteriosus in genetically altered *mgb* mice represents a structural defect in the target cell type necessary for normal physiological closure.

### Long-range enhancers

The genetic distance of the chromosome 11 insertion site from the myocardin gene suggests that the mgb phenotype occurs as a result of a position effect mutation. A wide range of important position effect mutations have been described [7], one of the most commonly proposed mechanisms being the disruption of a long-range enhancer element. The complex insertion/translocation defect characterized in *mgb* ^*−/−*^ mice involves the loss of a 26-kb segment of chromosome 11 during the 1-mb insertion of a transcriptionally active region chromosome 16. Either of these genetic events could have easily disrupted a long-range enhancer element critical to the normal temporal and spatial expression of myocardin. A similar loss of positive acting long-range enhancer elements has been shown to lead to a variety of human genetic diseases including Van Buchem disease, Leri–Weill dyschondrosteosis, Saethre–Chotzen syndrome, hypoparathyroidism, Rieger syndrome, Greig cephalopolysyndactyly, and X-linked deafness [8–14]. Each of these defects results from a tissue-specific gene dosage effect that occurs from the deletion or distal translocation of long-range cis-acting regulatory elements. These observations suggest that the organ-specific defects observed in the *mgb* ^−/−^ mouse result from a gene dosage effect associated with the interruption of a tissue-specific, long-range, cis-acting enhancer element located on chromosome 11 upstream from the myocardin transcriptional start site.

In summary, characterization of the genetic defects associated with the mgb phenotype has led to the discovery of a novel long-range regulatory element that is critical in modulating the tissue-specific expression of myocardin. Many key developmental control genes, like myocardin, appear to be regulated by overlapping enhancer activity, suggesting that gene dosage plays an important role in modulating the functional activity of these genes [7]. Long-range enhancers can be found in almost any genetic domain (introns, embedded within other gene promoters, etc.) and their activity can be modified by a single base mutation [7]. In addition, most long-range enhancer defects appear less severe than those directly associated with the gene transcript. These observations suggest that long-range enhancers might play a subtle but important role in many common diseases, making them attractive targets for SNPs or CNVs that appear spatially dissociated from their target gene.

## Renal pathogenesis

The *mgb* ^*−/−*^ mouse model of CON displays a highly orchestrated adaptive response that is designed to prevent permanent renal injury and permit rapid morphological and functional recovery. This model of renal adaptation appears to involve a balance between transforming growth factor beta (TGFβ)-directed pathogenesis, retinoic acid (RA)-mediated remodeling/repair, and steroid hormone modulation.

### Renal response to injury

Over half of the top 20 canonical pathways identified in affected *mgb* ^*−/−*^ kidneys involved renal response to injury, with the most activated upstream regulator being the TGFβ pathway [15]. This finding confirms prior morphological and biochemical studies in affected *mgb* ^*−/−*^ kidneys showing expanded TGFβ1 and connective tissue growth factor expression, increased density of α-smooth muscle actin-positive myofibroblasts, and the development of renal fibrosis [16]. These observations are highly consistent with current literature and highlight the key role that TGFβ plays in modulating progressive renal injury and fibrosis in a variety of kidney injury models including CON [17–19].

### Retinoic acid signaling

The role of RA in kidney development has been well characterized [20, 21]. We hypothesize that these same developmental functions are recapitulated during renal pathogenesis as a transient repair mechanism. This hypothesis is consistent with prior observations indicating that the *mgb* ^*−/−*^ kidneys show delayed maturation following birth [16]. In addition, retinoic acid has been shown to promote cell survival, antagonize the development of renal fibrosis, and mediate urothelial differentiation [22–24].

### Steroid hormone metabolism

The most significantly up-regulated mRNA detected in affected *mgb* ^−/−^ kidneys is corticosteroid-binding globulin (*Cbg*), which encodes the major transport protein for glucocorticoids and progestins in the blood. *Cbg* expression in the developing and postnatal kidney is highly regulated at both the mRNA and protein levels, and increases in the local concentration of glucocorticoid/progestin would be predicted to dampen the inflammatory response [25]. These observations are highly consistent with the fact that affected *mgb* ^−/−^ kidneys display limited inflammatory infiltrates during renal adaptation [16].

### Gender-specific responses

The most inhibited upstream pathway observed in *mgb* ^*−/−*^ kidneys involved histone deacetylases (Hdacs). Hdacs are a class of enzymes that remove acetyl groups from histones, permitting tight DNA packaging that often results in the down-regulation or inactivation of gene transcription [26]. Prior studies have shown that androgen, estrogen, and glucocorticoid receptors are substrates/binding partners for various members of the Hdac family, and that steroid hormone expression can influence perinatal programming [27]. This observation is intriguing, since affected male *mgb* ^*−/−*^ kidneys misexpress 18 sexually dimorphic gene targets, resulting in the down-regulation of 12 male-specific transcripts and up-regulation of 6 female-specific transcripts [15]. Epidemiological and experimental data support the concept that female gender is protective for some forms of renal disease [28–34]. Therefore, the expression of a more “female” transcriptome in affected male *mgb* ^*−/−*^ kidneys may initiate a transient cytoprotective genetic program that supports kidney remodeling and repair. Taken together, these observations suggest that steroid hormones play a complex role in modulating renal adaptation by suppressing acute inflammation and/or modifying the genetic control of cellular differentiation. These data provide a potential mechanism for gender-based differences in renal pathogenesis and identifies targets for the development of novel therapeutics in patients with CON.

### Renal urothelium

Comparison of *mgb* ^*−/−*^ transcriptomes with varying degrees of hydronephrosis identified an urothelial gene expression signature associated with severe obstruction [15]. The renal urothelium also showed alterations in organization and increased proliferation in affected kidneys. These changes in urothelial morphology represent some of the earliest detectable pathogenic events in affected *mgb* ^*−/−*^ kidneys, suggesting that the renal urothelium may play a key role in initiating the early remodeling/repair signals during renal pathogenesis. We postulate that these changes in urothelial morphology represent an early adaptive response to progressive renal injury that initiates a localized and reversible RA-based repair mechanism. In the face of continued renal insult, these early remodeling/repair responses may become exacerbated and irreversible resulting in permanent kidney damage and altered renal function.

**Fig. 2:**
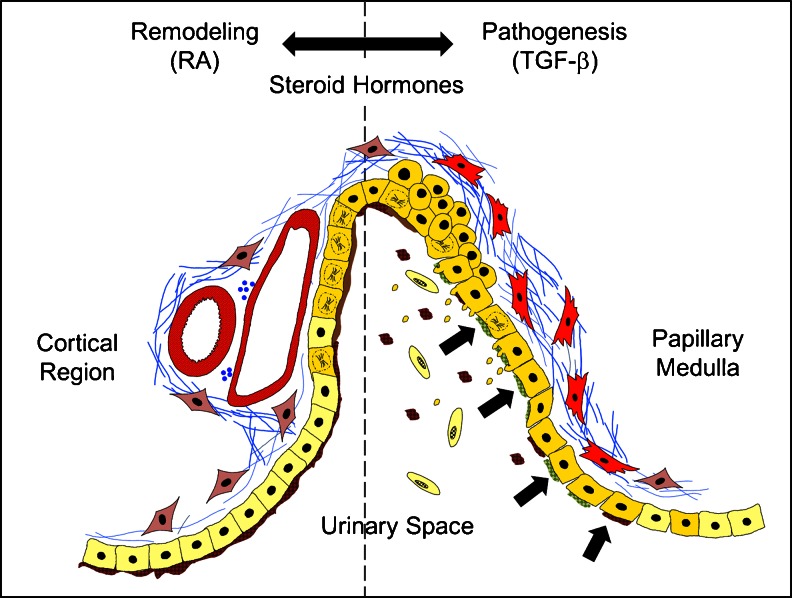
Model of renal adaptation and pathogenesis

In summary, CON in *mgb* ^−/−^ mice results in a progressive increase in back pressure within the renal pelvis, causing a gradual expansion of the renal urothelium (Fig. 2). Pelvic expansion triggers an RA-mediated response that results in renal urothelial cell proliferation from morphologically well-defined regions that are associated with large neurovascular bundles. This association provides direct access for neurohumoral input/output that may be important in modulating the pathogenic response in the contralateral kidney as well as other organ systems. Proliferating urothelial cells also display a less mature cellular phenotype that results in altered cell-to-cell interactions and apical plaque composition [15]. Although these changes may have short-term deleterious effects on membrane permeability and function, they most likely are critical in initiating activation of submucosal myofibroblasts resulting in collagen deposition immediately underlying the renal urothelium—one of the first major histopathological finding observed in *mgb* ^−/−^ kidneys [16].

Further progression of renal injury appears to be associated with a “second hit” on the affected kidney. This hypothesis is supported by the fact that longevity in female *mgb* ^−*/*−^ mice is relatively normal even in the face of chronic hydronephrosis [16]. In contrast, the majority of male *mgb* ^−*/*−^ mice die by 5–6 weeks of age as a result of several confounding processes that include transient development of acute high-pressure obstruction, urolithiasis, or ascending infection and pyelonephritis [16, 35]. Each of these events drives the molecular balance away from renal remodeling/repair toward expanded TGFβ–mediated renal pathogenesis. Under these conditions, there is increased recruitment of inflammatory cells and interstitial myofibroblasts, which can result in the development of severe interstitial fibrosis/scarring, the loss of renal tubules and glomeruli, reduced renal function, and eventually end-stage renal disease (ESRD) [35]. It is intriguing to postulate that the changes induced during renal adaptation alter the functionality of the urothelium, thereby increasing the kidney’s susceptibility to further disease progression. For example, altered apical plaque content during urothelial proliferation may increase the kidneys’ susceptibility to infection. In a similar manner, predetermined genetic susceptibilities, as well as environmental exposures, may also play key roles in exacerbating or attenuating renal pathogenesis.

## Conclusions: lessons learned from the *mgb*^*−/−*^ mouse

Animal models provide a unique window into the complex pathophysiological responses associated with the development and progression of disease. The mgb mouse model of CON provides several unique insights into the mechanisms associated with lower urinary tract development and pathogenesis. First, genetic defects associated with long-range regulatory domains have the potential to alter the level of gene expression in a temporal and spatially specific manner. Standard genetic approaches to identifying disease-specific loci may overlook these regulatory domains, since they often occur at great distances from their given gene target. The mgb mouse highlights the importance of long-range transcriptional regulatory domains in modulating quantitative trait loci, and suggests that these “hidden” genetic elements might play a key role in many human diseases.

Second, the *mgb* ^*−/−*^ mouse model of CON demonstrates that a chronic, low-pressure obstruction results in significant renal remodeling that may in turn prime the kidney for continued disease progression if left untreated. Similar models of disease adaptation and progression are observed in a variety of other organ systems. For example, cardiac remodeling following the development of hypertension results in left ventricular hypertrophy, increased cardiac risk, and the potential, if left untreated, for organ failure. These similarities suggest a common functional paradigm in organ pathogenesis and that early surgical intervention might be warranted in the treatment of CON.

## Abbreviations

CON: Congenital obstructive nephropathy
mgb: Megabladder
RA: Retinoic acid
TGFβ: Transforming growth factor beta
ESRD: End-stage renal disease
Cbg: Corticosteroid-binding globulin
Hdac: Histone deacetylase
